# Noninvasive Ultrasound Stimulation of Ventral Tegmental Area Induces Reanimation from General Anaesthesia in Mice

**DOI:** 10.34133/2021/2674692

**Published:** 2021-04-12

**Authors:** Tianyuan Bian, Wen Meng, Meihong Qiu, Zhigang Zhong, Zhengrong Lin, Junjie Zou, Yibo Wang, Xiaowei Huang, Lisheng Xu, Tifei Yuan, Zhili Huang, Lili Niu, Long Meng, Hairong Zheng

**Affiliations:** ^1^Institute of Biomedical and Health Engineering, Shenzhen Institutes of Advanced Technology, Chinese Academy of Sciences, 1068 Xueyuan Avenue, Shenzhen, China 518055; ^2^Department of Pharmacology, School of Basic Medical Sciences, Fudan University, Shanghai, China 200032; ^3^College of Medicine and Biological Information Engineering, Northeastern University, 195 Innovation Road, Shenyang 110016, China; ^4^Shanghai Mental Health Center, Shanghai Jiaotong University School of Medicine, Shanghai, China 200030

## Abstract

Evidence in animals suggests that deep brain stimulation or optogenetics can be used for recovery from disorders of consciousness (DOC). However, these treatments require invasive procedures. This report presents a noninvasive strategy to stimulate central nervous system neurons selectively for recovery from DOC in mice. Through the delivery of ultrasound energy to the ventral tegmental area, mice were aroused from an unconscious, anaesthetized state in this study, and this process was controlled by adjusting the ultrasound parameters. The mice in the sham group under isoflurane-induced, continuous, steady-state general anaesthesia did not regain their righting reflex. On insonation, the emergence time from inhaled isoflurane anaesthesia decreased (sham: 13.63 ± 0.53 min, ultrasound: 1.5 ± 0.19 min, *p* < 0.001). Further, the induction time (sham: 12.0 ± 0.6 min, ultrasound: 17.88 ± 0.64 min, *p* < 0.001) and the concentration for 50% of the maximal effect (EC50) of isoflurane (sham: 0.6%, ultrasound: 0.7%) increased. In addition, ultrasound stimulation reduced the recovery time in mice with traumatic brain injury (sham: 30.38 ± 1.9 min, ultrasound: 7.38 ± 1.02 min, *p* < 0.01). This noninvasive strategy could be used on demand to promote emergence from DOC and may be a potential treatment for such disorders.

## 1. Introduction

Disorders of consciousness (DOC) involve the absence or severe loss of consciousness and include comas, vegetative states, and minimal consciousness states. DOC are most often caused by acute brain injuries such as hypoxic/ischemic neurological injury or traumatic brain injury (TBI) [1, 2]. However, no valid data-based clinical guidance exists currently to direct physicians in the treatment of patients with DOC. A growing body of evidence from both the neural circuit and system levels has indicated that arousal pathways play an instrumental role in emergence from DOC. In addition, recent reports have indicated that activation of cholinergic, monoaminergic, dopaminergic, noradrenergic, and histaminergic arousal pathways can lead to emergence from DOC [3–7]. Therefore, neuromodulation techniques promise to become potential experimental therapeutic tools for DOC.

Schiff et al. demonstrated that bilateral deep brain electrical stimulation (DBS) of the central thalamus in DOC patients can alter behavioral responsiveness [8]. However, DBS is invasive and associated with complicated surgery. Xia et al. reported that rehabilitation of DOC patients can be aided by 10 Hz transcranial magnetic stimulation of the left dorsolateral prefrontal cortex [9]. In addition, Martens et al. indicated that 4 weeks of transcranial direct current stimulation modestly improved recovery of consciousness signs in patients with chronic minimally conscious states [10]. However, the spatial resolution and penetration depth of the above technologies could be further improved. Accordingly, there is a need to develop other new tools to facilitate emergence from DOC, and these tools should satisfy multiple requirements: they should be noninvasive, dynamic, and able to penetrate deep into the brain.

As ultrasound can penetrate the skull noninvasively in a focused manner, it is a potentially potent neurostimulation tool [11–13]. Animal and human studies have suggested that ultrasound (US) stimulation at acoustic intensities similar to or lower than those typically used in ultrasound diagnosis can excite and inhibit neural activity [14, 15]. Previous studies by our group and others have indicated that US stimulation can evoke action potentials in brain slices [16–18]. Transcranial focused ultrasound can be used to modulate neural activity transiently, reversibly, reproducibly, and focally [12, 19], even in deep brain areas such as the amygdala and anterior cingulate cortex of macaques [20]. Furthermore, focused transcranial ultrasound can noninvasively modulate cortical or deep brain areas in humans [21, 22]. Even more excitingly, Yoo et al. found that US stimulation of the thalamus of ketamine/xylazine anaesthetized rats reduced the time to emergence from the anaesthesia [23]. These results suggest that ultrasound can penetrate the skull and may have potential for clinical application in awakening patients from DOC. The ventral tegmental area (VTA), an important component of the arousal loop in the brain, secretes large amounts of dopamine (DA), which is an important excitatory neurotransmitter in arousal behavior [24]. However, whether US stimulation of the VTA can elicit the transition from an unconscious, anaesthetized state to a waking state remains unknown.

**Figure 1:**
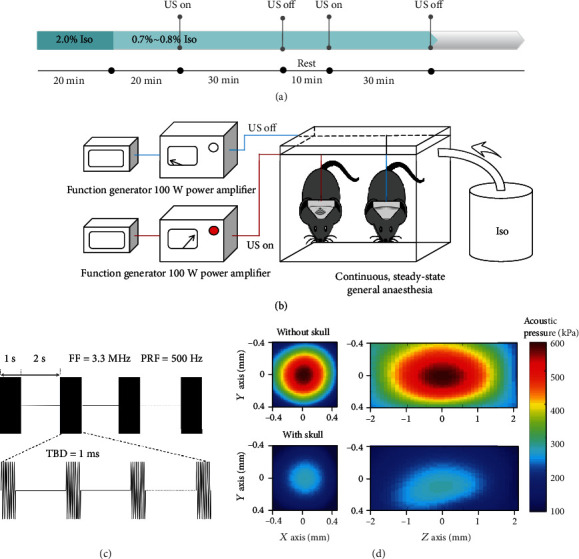
Experimental design and ultrasound parameters. (a) Arousal experiment process. Mice received 2.0% isoflurane by inhalation for 20 min to induce deep anaesthesia. Then, the isoflurane dose was adjusted to 0.7%–0.8%, which maintained the LORR. This isoflurane dose was continued until the end of the experiment. Ultrasound stimulation was initiated after 40 min of anaesthesia and continued for two doses of 30 min or until the righting reflex behavior returned. (b) Schematic diagram of ultrasound-induced reanimation from general anaesthesia. (c) Ultrasound stimulation parameters: PRF = 500 Hz, TBD = 1 ms, SD = 1 s, DC = 50%, and ISI = 2 s. (d) Acoustic pressure distributions in the transverse and longitudinal planes in the absence and presence of mouse skull bone.

This study was aimed at inducing emergence from general anaesthesia using US stimulation of the VTA to reduce the incidence of delayed emergence. Firstly, mice were subjected to continuous, steady-state general anaesthesia (CSSGA) with isoflurane. A wearable ultrasound transducer with a millimeter-scale focus was manufactured and used to stimulate the VTA neurons. Transcranial ultrasound (3.3 MHz fundamental frequency, 50% duty cycle (DC), 500 Hz pulse repetition frequency (PRF), 1 ms tone burst duration (TBD), 1 s sonication duration (SD), and 2 s interstimulus interval (ISI)) was applied to the VTA (Figure 1). The experiments demonstrated that US stimulation of the VTA observably reduced the latency of mice to recover their righting reflex, prolonged the induction time for isoflurane anaesthesia, and increased the effective anaesthetic concentration for 50% loss of the righting reflex. Our results also indicated that the D1 antagonist SCH-23390 can inhibit the arousal response caused by ultrasound stimulation, which is not the case with the D2 antagonist (s)-(-)-sulpiride. These results suggest that ultrasound-activated DA release in the VTA induces emergence from isoflurane anaesthesia through a D1 receptor-mediated process and generates arousal proof of both behavior and neurophysiology. In addition, we found that US stimulation of VTA can promote emergence in TBI mice. Therefore, ultrasound may provide a new method of treating patients with DOC.

## 2. Results

### 2.1. Ultrasound Stimulation Increases the Expression of c-Fos in the VTA

**Figure 2:**
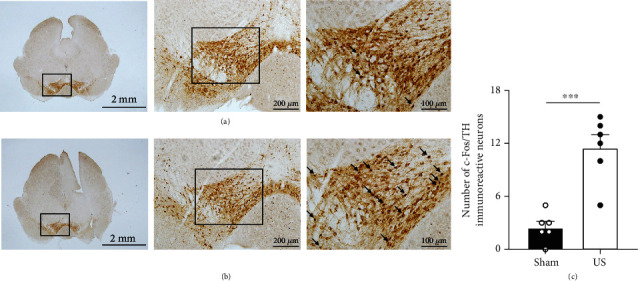
Ultrasound stimulation increases the abundance of c-Fos-positive neurons in the VTA. Representative c-Fos expression of the VTA in (a) the sham group and (b) the US group. Double labelling of TH (brown) and c-Fos (black) in the VTA. Arrows indicate c-Fos-positive cells. (c) Number of c-Fos-positive cells in the US group, showing a significant increment compared to that in the sham group. c-Fos-positive cells costained with dopamine neurons were counted in the entire VTA region of the brain slice (*n* = 6, mean ± SEM, ^∗∗∗^*p* < 0.001, independent-sample *t*-test).

Ultrasound-induced VTA neuronal activation can be quantitatively assessed by c-Fos immunohistochemistry [25]. To determine the VTA activity, we randomly divided the mice into a US group (*n* = 6) and a sham group (*n* = 6). Unlike the sham group, the US group was given 40 min of US at an acoustic pressure of 586 kPa. The perfused brains were sectioned at a thickness of 30 *μ*m and were doubly immunolabelled for c-Fos (black) and tyrosine hydroxylase (TH; brown). Significantly higher c-Fos expression in the VTA in the US group was observed than in the sham group (sham: 2.50 ± 0.67, US: 11.50 ± 1.48, *n* = 6, *p* < 0.001, 1 − *β* = 1.00, independent-sample *t*-test), as shown in Figure 2. More staining images of the VTA in the sham and US groups are provided in Fig. S1. We similarly observed a significant increase in c-Fos expression of the periaqueductal grey (PAG) and the locus coeruleus (LC), as shown in Fig. S2, S3.

### 2.2. Ultrasound Stimulation Induces Reanimation from General Anaesthesia

Our previous research indicated that the threshold for effective evoked action potential release by ultrasound is 0.25 MPa in the hippocampal slice of mice [18]. Different acoustic pressures were used to activate neuronal activity. In the normal arousal experiments, the induction and maintenance of isoflurane-containing CSSGA were conducted following the protocol depicted in Figure 1(a). US stimulation of the VTA was initiated at different acoustic pressures with the absence of the mouse skull (360 kPa, 586 kPa, and 758 kPa) to test behavioral arousal in response to ultrasound.

**Figure 3:**
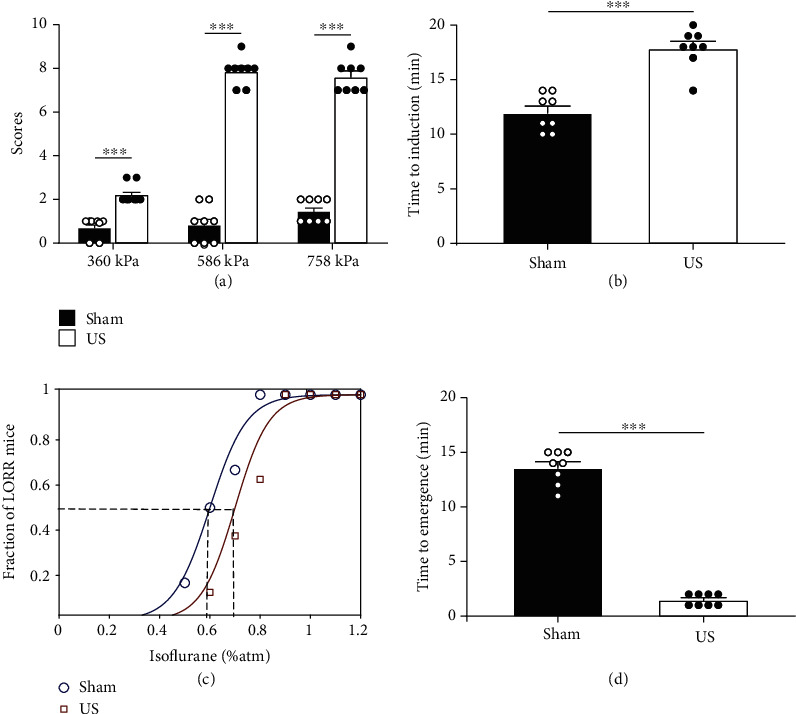
Effects of ultrasound stimulation of the VTA on isoflurane sensitivity, induction, and emergence time. (a) Behavioral scores of the sham and US groups at different acoustic pressures (without skull). Compared to the sham group, the US group has a much higher score (*n* = 8, mean ± SEM, ^∗∗∗^*p* < 0.001, independent-sample *t*-test). (b) Ultrasound stimulation of the VTA delays the time to anaesthesia induction in mice. The time of descent into anaesthesia is significantly prolonged in the US group compared with the sham group (*n* = 8, mean ± SEM, ^∗∗∗^*p* < 0.001, independent-sample *t*-test). (c) Dose-response curves of mice in the US group (red line) and sham group (black line), showing the progressive increment in the proportion of mice that lost the righting reflex as a function of isoflurane concentration. The EC50 is larger for the mice in the US group (squares) than for the sham mice (circles). The horizontal axis is the dose of isoflurane as a percentage of the atmosphere concentration (%atm) (*n* = 8). (d) Emergence time from exposure to 1% isoflurane of the sham and US groups (*n* = 8, mean ± SEM, ^∗∗∗^*p* < 0.001, independent-sample *t*-test).

The arousal scoring system was developed by referencing and refining methods from previous studies [26, 27]. During US stimulation with continuous inhalation of 0.7–0.8% isoflurane, the frequencies of the head, whisker, leg, and tail movements were analyzed and classified as none, mild, and moderate and scored as 0, 1, and 2, respectively. A score of 2 was assigned if a mouse returned to all four paws on the ground, and 0 was assigned if it remained lying on its side. The total score was the sum of the above item scores, with a maximum possible score of 10. The integral protocol of the normal behavioral experiment is shown in Supplementary Video S1. The results demonstrated that US stimulation dramatically improved the state of awareness in contrast to the sham group at an acoustic pressure of 360 kPa (sham: 0.75 ± 0.16, ultrasound: 2.25 ± 0.16, *n* = 8, *p* < 0.001, 1 − *β* = 0.99, independent-sample *t*-test, Table S1). Similar trends were also found at acoustic pressures of 586 kPa (sham: 0.88 ± 0.30, ultrasound: 7.88 ± 0.23, *n* = 8, *p* < 0.001, 1 − *β* = 1.0, independent-sample *t*-test, Table S2) and 758 kPa (sham: 1.5 ± 0.19, ultrasound: 7.63 ± 0.26, *n* = 8, *p* < 0.001, 1 − *β* = 1.0, independent-sample *t*-test, Table S3), as shown in Figure 3(a). At an acoustic pressure of 360 kPa, none of the mice (0/8) recovered their righting reflex during the experiment. However, 75% of the mice (6/8) regained this reflex at an acoustic pressure of 586 kPa, and 62.5% of the mice (5/8) did so at an acoustic pressure of 758 kPa. Consequently, ultrasound stimulation with an acoustic pressure of 586 kPa was used in the subsequent experiment to ensure the validity and safety of the treatment.

Then, it was investigated whether ultrasound stimulation could modify the onset timing of isoflurane-induced anaesthesia. The anaesthesia induction time of the US group was significantly increased compared with that of the sham group (sham: 12.0 ± 0.60 min, ultrasound: 17.88 ± 0.64 min, *n* = 8, *p* < 0.001, 1 − *β* = 0.99, independent-sample *t*-test), as shown in Figure 3(b). Next, the concentration for 50% of maximal effect (EC50), which means the concentration at which half of the mice lost their righting reflex due to anaesthesia, was investigated [28]. We used increasing doses of isoflurane to find EC50 and examine whether the anaesthetic effects of the substance were altered by US stimulation. The effect of anaesthesia depended on the dose of anaesthetic. However, unlike the sham group, the mice that received US stimulation exhibited decreased sensitivity to isoflurane. The concentration at which half of the mice showed a loss of righting reflex (LORR) was approximately 0.6% and 0.7% in the sham and ultrasound groups (*n* = 8), respectively, as shown in Figure 3(c). We further examined whether emergence from isoflurane anaesthesia changed in US mice. When the mice were returned to room air after exposure to 1% isoflurane for 30 min, the US group had a shorter latency (emergence time) to recover the righting reflex than the sham group (sham: 13.63 ± 0.53, ultrasound: 1.5 ± 0.19 min, *n* = 8, *p* < 0.001, 1 − *β* = 0.99, independent-sample *t*-test), as shown in Figure 3(d).

### 2.3. Ultrasound-Induced Reanimation Is Inhibited by the D1 Antagonist SCH-23390

**Figure 4:**
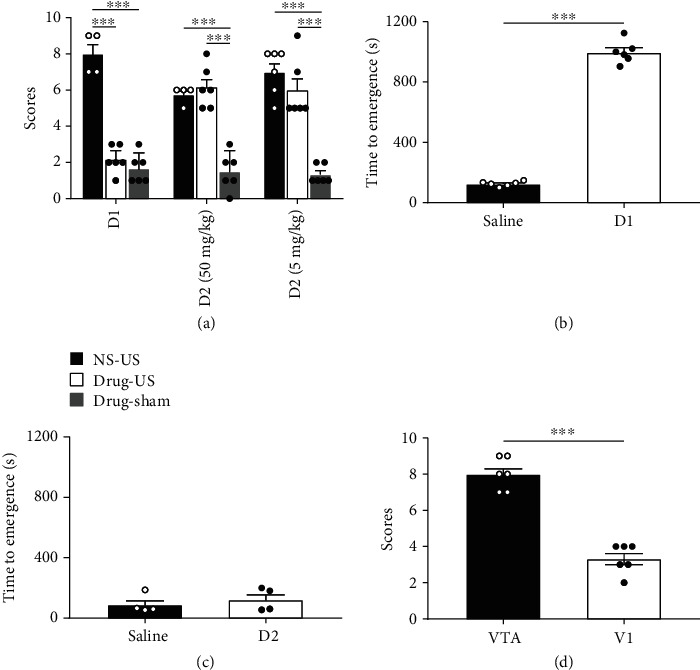
Mechanism of ultrasound stimulation in reanimation from general anaesthesia. (a) Behavioral scores of the normal saline and drug groups (*n* = 4 for NS-US group, *n* = 6 for rest of the groups, mean ± SEM, ^∗∗∗^*p* < 0.001, *p* > 0.05, ANOVA). (b) Emergence time of the D1 receptor antagonist group and saline group (*n* = 4, mean ± SEM, ^∗∗∗^*p* < 0.001, independent-sample *t*-test). (c) Emergence time of the D2 receptor antagonist group (50 mg/kg) and saline group (*n* = 4, mean ± SEM, *p* > 0.05, independent-sample *t*-test). (d) Behavioral scores during ultrasound stimulation of the VTA and visual cortex (*n* = 6, mean ± SEM, ^∗∗∗^*p* < 0.001, independent-sample *t*-test).

Dopamine (DA) is a familiar arousal-inducing substance, and previous studies have shown that the dopamine D1 receptor is involved and important in anaesthesia arousal [26]. To evaluate whether antagonizing D1-like receptors inhibits ultrasound-induced arousal from an unconscious, anaesthetized state, we treated mice with or without the antagonist of the D1 receptor SCH-23390. The mice that received intraperitoneal injections of SCH-23390 (0.1 mg/kg, i.p.) had significantly lower scores than the normal saline ultrasound (NS-US) group during US (NS-US: 8.0 ± 0.58, *n* = 4; SCH-23390 ultrasound: 2.17 ± 0.31, *n* = 6; SCH-23390 sham: 1.67 ± 0.33, *n* = 6; NS-US VS SCH-23390 ultrasound: *p* < 0.001; NS-US VS SCH-23390 sham: *p* < 0.001; ANOVA), as shown in Figure 4(a). Figure 4(b) demonstrates that the emergence time is prolonged after injection of the D1 receptor antagonist (SCH-23390 group: 997.67 ± 30.25 s, saline group: 125.17 ± 6.74 s, *n* = 4, *p* < 0.001, 1 − *β* = 1.00, independent-sample *t*-test).

In addition, we used a D2 receptor antagonist ((s)-(-)-sulpiride, 50 mg/kg and 5 mg/kg, i.p.) to verify the role of the D2 receptor in the anaesthesia arousal experiments. The results showed that the scores of mice treated with US stimulation were significantly higher than those of the mice in the D2-sham group and no significant difference between the D2 ultrasound and NS-US groups (NS-US: 5.75 ± 0.25, *n* = 4; 50 mg/kg sulpiride ultrasound: 6.17 ± 0.48, *n* = 6; 50 mg/kg sulpiride sham: 1.5 ± 0.43, *n* = 6; NS-US vs. 50 mg/kg sulpiride sham: *p* < 0.001; 50 mg/kg sulpiride ultrasound vs. 50 mg/kg sulpiride sham: *p* < 0.001; NS-US: 7.00 ± 0.52; 5 mg/kg sulpiride ultrasound: 6.00 ± 0.68; 5 mg/kg sulpiride sham: 1.33 ± 0.21, *n* = 6; NS-US vs. 5 mg/kg sulpiride sham: *p* < 0.001; 5 mg/kg sulpiride ultrasound vs. 5 mg/kg sulpiride sham: *p* < 0.001; ANOVA). Figure 4(c) shows that the emergence time does not significantly differ between the D2 receptor antagonist and saline groups (50 mg/kg sulpiride group: 123.25 ± 38.47 s, saline group: 90.75 ± 31.84 s, *n* = 4, *p* > 0.05, 1 − *β* = 0.14, independent-sample *t*-test). These results demonstrate that D1 receptors play a major role in US-stimulated reversal of anaesthesia, whereas the D2 receptors do not affect arousal behavior (Table S4, S5; Video S2, S3). In addition, we repeated the arousal experiment with US stimulation of the primary visual cortex (V1). Figure 4(d) indicates that one of the six mice (1/6) recovered the righting reflex during US stimulation of V1 (VTA: 8.00 ± 0.37, V1: 3.33 ± 0.33, *n* = 6, *p* < 0.001, 1 − *β* = 0.99, independent-sample *t*-test, Table S6, Video S4).

### 2.4. Ultrasound Stimulation Induces Reanimation in TBI Model Mice

**Figure 5:**
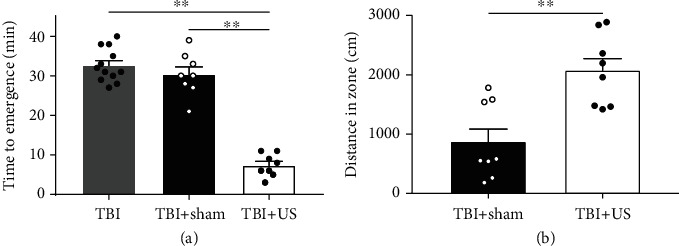
Behavioral experiment results. (a) Time of emergence, which is significantly decreased for the mice in the US group (*n* = 12 for TBI group, *n* = 8 for TBI+sham and TBI+US groups, mean ± SEM, ^∗∗^*p* < 0.01, independent-sample *t*-test). (b) Performance of mice, which is significantly improved in the US group (*n* = 8, mean ± SEM, ^∗∗^*p* < 0.01, independent-sample *t*-test).

We further investigated the clinical application of ultrasound neuromodulation in mice with TBI. The mouse model of TBI was prepared according to the method described in reference [29]. The effect of US stimulation was evaluated using the emergence time from general anaesthesia and an open field test.  Figure 5(a) reveals that the mice in the US group have a significantly decreased time of emergence (TBI: 32.67 ± 1.22 min, *n* = 12; TBI+sham: 30.38 ± 1.93 min, *n* = 8; TBI+US: 7.38 ± 1.02 min, *n* = 8; TBI vs. TBI+US: *p* < 0.01, 1 − *β* = 1.00; TBI+sham vs. TBI+US: *p* < 0.01, 1 − *β* = 1.00, independent-sample *t*-test). Meanwhile, in the open field test, the motor function of mice in the US group exhibits significant improvement (TBI+sham: 877.57 ± 228.52 cm; TBI+US: 2078.65 ± 212.26 cm, *n* = 8, *p* < 0.01, 1 − *β* = 0.87, independent-sample *t*-test), as shown in Figure 5(b).

### 2.5. Safety of Ultrasound Stimulation

**Figure 6:**
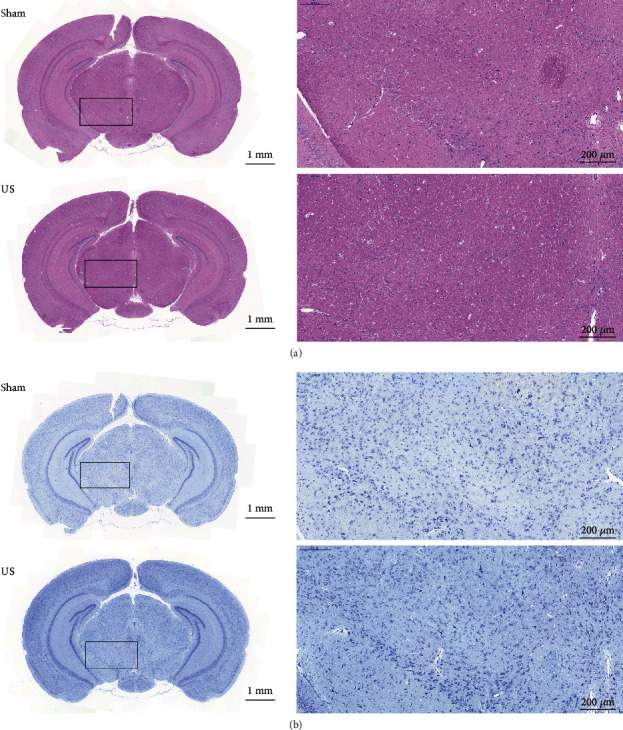
Histological evaluations of the VTA with and without ultrasound stimulation. (a) Representative H&E staining and (b) Nissl staining of sections from the sham and US groups. The results show no abnormalities (haemorrhaging, tissue damage, or neuron loss) in the VTA.

Haematoxylin and eosin (H&E) and Nissl staining were performed to assess the safety of ultrasound stimulation of mice (*n* = 8 for ultrasound; *n* = 6 for sham).  Figure 6(a) shows H&E-stained brain slices from each group. Compared with the sham group, there are no abnormal findings (haemorrhaging or tissue damage) in the VTA of the US group after stimulation. As illustrated by the Nissl staining results in Figure 6(b), the neuronal density through the brain is similar between groups. Therefore, transcranial ultrasound stimulation may safely be used for reanimation from general anaesthesia.

## 3. Discussion

In this paper, we indicated that ultrasound stimulation of VTA induced the arousal response in general anaesthesia and TBI mice. The arousal response is greatly diminished by the methodical delivery of the D1 antagonist SCH-23390 prior to US stimulation, whereas intraperitoneal injection of D2 antagonist (s)-(-)-sulpiride did not inhibit arousal in mice. This result suggests that downstream mechanisms mediated by the involvement of D1 receptors are mostly responsible for the ultrasound activation of VTA neurons during CSSGA. In addition, we found that the ultrasound stimulation of VTA in mice with TBI has remarkable arousal effects. Therefore, ultrasound may be a useful tool in DOC treatment.

The VTA is a crucial structure for motivated behaviors and includes dopaminergic, glutamatergic, and GABAergic neurons [30]. The bursting activity of VTA dopaminergic neurons significantly increases during rapid eye movement sleep [31]. Further investigation showed that the activity of VTA dopaminergic neurons was reduced during nonrapid eye movement sleep [32]. These studies reveal that VTA dopaminergic neurons were important participants in the sleep/wake system. Moreover, electrical stimulation of the VTA induced an arousal response during general anaesthesia with isoflurane [27]. Taylor et al. demonstrated that activation of VTA dopaminergic neurons induced wakefulness from 0.8 to 0.9% isoflurane anaesthesia and that the D1 receptors were involved in inducing wakeful behavior [26]. The hypothesis that dopamine release from VTA neurons is involved in awakening from general anaesthesia is substantiated by these results. In the present study, D1 and D2 antagonists were used to test the hypothesis that the emergence from isoflurane anaesthesia is caused by the activation of dopaminergic neurons. The arousal response was reduced by intraperitoneal injection of the D1 antagonist into the mice before US stimulation, whereas D2 antagonist (s)-(-)-sulpiride had no significant effect on the arousal response. These findings suggest that the D1 receptor is important for promoting emergence from anaesthesia induced by ultrasound stimulation.

In the last decade, many experimental techniques have emerged to study the neural circuits underlying emergence from general anaesthesia in rodents, including genetic manipulations [33], microdialysis [34], targeted brain lesions [35], electrical stimulation [27], and optogenetics [26]. Ultrasound as a novel and improved neuromodulation method can be utilized to investigate anaesthetic-induced unconsciousness and restored consciousness. Compared with electrical stimulation and optogenetics, ultrasound stimulation can be administered noninvasively through the skull, focused at any site within the brain, and used concurrently with imaging techniques (i.e., functional MRI) [21]. Interestingly, the arousal response induced by ultrasound stimulation takes longer to develop than the arousal effects of VTA electrical stimulation or optogenetics [26, 27]. One possible explanation is that a relatively small proportion of DA neurons in the VTA is experimentally triggered by ultrasound stimulation, as opposed to electrical stimulation, which involves 100% recruitment. The other possible explanation is a difference in the modulatory mechanism [36]. In addition, we found that ultrasound stimulation of the VTA activated the neurons of the LC, which can be considered to be caused by arousal behavior, as the LC area is involved in the sleep-wake function (Fig. S2) [37–39]. The expression of c-Fos within the PAG area significantly differs between the sham and US groups (Fig. S3). The potential reasons could be as follows: (1) The thresholds at which various neurons were activated by ultrasound differed between brain areas. Thus, the thresholds for neuronal activation in different brain areas should be studied further. (2) There is evidence that activation of the VTA can inhibit sustained nociceptive transmission by a mechanism related to VTA projections to GABAergic nerve fibers in the PAG region [40, 41]. Experiments have shown that there is a cardiovascular inhibitory pathway from the VTA to the PAG area [42]. These evidences demonstrated the existence of a pathway for VTA projection to the PAG region, which may also contribute to the expression of c-Fos in the PAG area. However, the PAG region is related to pain, aggression, etc. Therefore, the emergence induced by ultrasound may be mainly attributed to the activation of the VTA.

Ultrasound waves induce mechanical, cavitation, and thermal effects on biological tissues. Although few details are known about the mechanism of ultrasound-mediated neuromodulation, it has been reported that the neurophysiology of local neural circuits can be significantly affected by ultrasound. [43]. *C. elegans* neural circuits can be activated by low-intensity US stimulation which amplified the mechanical deformation by microbubbles, and the mechanosensitive ion channel TRP-4 is involved in ultrasound-altered neuronal activity. The misexpression of this channel affects ultrasound-induced neuronal activity changes. [44]. The *C. elegans* response to ultrasound pulses requires expression of the DEG/ENaC ion channel MEC-4 because the absence of the ion channel MEC-4 attenuates its response to ultrasound stimulation [45]. In addition, US stimulation of the sodium and potassium mechanosensitive ion channels (Na_V_1.5; TREK-1, TREK-2, and TRAAK) modulates the currents through the channels in *Xenopus* oocytes [46]. Recently, Ye et al. reported that mechanosensitive channel MscL can be switched on by ultrasound waves to regulate neuronal activities [17]. Collectively, one of the mechanisms of ultrasound stimulation is the activation of the mechanosensitive ion channels of cellular membranes. Because of the multiple effects of ultrasound, evaluating the safety of transcranial focused ultrasound is necessary. Mounting evidence has shown that low-intensity pulsed ultrasound is safe for transcranial stimulation of mice and rats [47–49]. Low-intensity pulsed ultrasound stimulation was used on Alzheimer's disease rats to reverse aluminium-induced cerebral damage [50], and transcranial pulse ultrasound was used on the motor cortex and intact hippocampus [13]. Our previous studies have indicated that the primary cilia of rat hippocampal neurons can be adjusted by ultrasound [51] and that stimulation can also improve motor deficits in Parkinsonian mice [47]. These results demonstrate that transcranial focused ultrasound provides a safe method of stimulating the brain. The ultrasound intensity used in this study was well below the regulatory limit for nonobstetric ultrasound imaging (maximum *I*_SPPA_ = 190 W/cm^2^), which is 7.94 W/cm^2^ (*I*_SPPA_) [52]. Moreover, the mechanical index (MI) of the present study was about 0.3, well within the ranges of safety guidelines. The most elevated temperature of brain tissue in this study was theoretically calculated to be only 0.1°C (Supplementary method) [53]. The surface temperature of the skull before ultrasound stimulation is 27.3°C (mice anaesthetized with isoflurane led to a lower temperature) and becomes 31.6°C after ultrasound stimulation (lower than normal body temperature) (Fig. S4). Collectively, the ultrasound parameters used in this experiment were in the ranges of the safety guidelines for clinical ultrasound imaging. No biological damage was discovered along the ultrasound propagation path in the brain (Figure 6).

Two recent reports argued that ultrasound neuromodulation requires auditory pathway activation in rodents [54, 55]. However, recent work by Niu et al. using chemical-deafened rodent models showed that the ultrasound brain modulation is confined by localized response without involving auditory networks [56]. The hearing range of mice is from 2.3 kHz to about 75 kHz [57]. The PRF of the ultrasound transducer used in this study was 500 Hz, which may not induce the auditory loop activation. In addition, one of the six mice (1/6) recovered the righting reflex during US stimulation of the visual cortex (Figure 4(d)). In another of our unpublished studies, we found that ultrasound stimulation could induce reanimation from anaesthesia through activating dopamine neurons in VTA. Furthermore, the mouse model of mechanical-induced deafness was prepared according to the method described in reference [58]. Ultrasound stimulation showed similar effects on both normal and deaf mice. These results may suggest that ultrasound directly activates the VTA rather than doing so through the auditory pathways.

As with all research, the present study has its limitations. For instance, it did not deeply explore the relationship between the arousal effect of ultrasonic stimulation and different types of anaesthesia, such as propofol. Hence, the arousal effects of different types of anaesthesia and different ultrasound stimulation parameters need to be explored in further studies. Moreover, the fundamental frequency of the ultrasonic transducer was 3.3 MHz. This parameter will be decreased to less than 650 kHz in subsequent experiments on primates and humans to allow for the attenuation of ultrasound as it passes through the skull [14, 59]. Further, the method for modeling comas in our study was simple and limited to comas caused by TBI. In addition, the approach utilized to evaluate wakefulness was only an open field test. Multiple DOC models should be added in subsequent studies to characterize the effects of US stimulation on other different types of comas. Simultaneously, additional means and methods of evaluation should be utilized to assess the mobility capacity, consciousness recovery, and other aspects.

This study demonstrated that ultrasound neuromodulation can safely induce rapid arousal from an anaesthesia state or acute comatose state by stimulating VTA. Our results provide a new application of ultrasound neuromodulation technology in neuroscience and a novel noninvasive procedure to accelerate recovery from general anaesthesia, which may be applied to alleviate or obviate emergence-related problems, including coma, postoperative delirium, cognitive dysfunction, and even circadian rhythm disorders.

## 4. Materials and Methods

### 4.1. Animal Preparation

The protocol of all animal experiments was approved by the Institutional Ethical Committee of Animal Experimentation of Shenzhen Institutes of Advanced Technology (Chinese Academy of Sciences), and they were conducted in accordance with the Laboratory Animal Guideline of Welfare and Ethics. The IACUC number is SIAT–IACUC–20190321–ZGKXYSZXJJSYJY-YB-ZHR-01-01. All mice used in this study were male C57BL/6J mice (Beijing Vital River Laboratory Animal Technology Co. Ltd., 8 weeks old, 22 g ± 10%). The animals were housed at 23°C ± 1°C and 55 ± 5% humidity with a 12 h/12 h light/dark cycle and had unfettered access to food and water.

All mice were anaesthetized with 2.0% isoflurane and placed in a transparent closed container (RWD, Shenzhen). The scalp of each mouse was removed with surgical scissors to expose its skull. At the same time, we selected the V1 area of the mouse as another region to demonstrate the specificity of US stimulation. Collimators to focus ultrasound to the VTA (related to the bregma: −3.40 mm anterior/posterior, −0.48 mm medial/lateral, and −4.30 mm dorsal/ventral) and V1 (related to the bregma: 1.10 mm anterior/posterior, −1.50 mm medial/lateral, and −0.50 mm dorsal/ventral) were mounted on the surface of the mouse skull with medical bone cement. After the dental cement had solidified, the mouse was removed from the stereotaxic apparatus and returned to its home cages. The experiments on the mice were started after a 7-day recovery period.

### 4.2. Ultrasound Stimulation Parameters

The radio frequency signals with a frequency of 3.3 MHz were generated by a four-channel function generator (DG4162, RIGOL, China) and amplified by a 100 W power amplifier (2100L, EI, USA) to drive an ultrasound transducer. The parameters used in this experiment were PRF of 500 Hz, pulse duration of 1 ms, duty cycle of 50%, sonication duration of 1 s, ISI of 2 s, and acoustic pressure of 360 kPa, 586 kPa, and 758 kPa. The acoustic pressures were measured in the absence of the skull. The original and transcranial acoustic fields were measured by a calibrated needle hydrophone (SN2791, 0.5 mm probe, Precision Acoustics, UK) in a degassed water tank using a 3D ultrasound intensity measurement system (UMS3, Precision Acoustics, UK). The attenuation of acoustic pressure due to propagation through the skull and dura of mice was about 54%. The transcranial focus width is 0.24 mm, and focus length is 1.45 mm at an acoustic pressure of 586 kPa. The focus width is 0.49 mm and the focus length is 3.05 mm at an acoustic pressure of 758 kPa.

The focus of the ultrasound transducer was visually observed by an ultrasound beam analyzer (Onda, USA). The acoustic field distributions of the transducer without and with the mouse skull are shown in Fig. S5.

The skull heating induced by ultrasound stimulation was measured using an infrared thermal imager (R300, NEC Avio, Tokyo, Japan). The temperature profile before and after the ultrasound stimulation was visualized.

### 4.3. Behavioral Tests

#### 4.3.1. CSSGA with Isoflurane

The gases in the anaesthesia vessel were constantly sampled by a light wave interference gas monitor (FI-8000, RIKEN KEIKI, Japan) in all experiments with isoflurane to ensure that the isoflurane concentration reached the experimental standard.

#### 4.3.2. Normal Arousal Experiment

The experimental schedule of ultrasound stimulation is shown in Figure 1(a). A mobile anaesthesia machine (RWDr520, RWD, China) was used to ensure the concentration of anaesthetics in the glass chamber. Each mouse was placed into the chamber and anaesthetized with isoflurane (2%) in the air for 20 min. A heating pad was placed under the chamber to maintain the body temperature of the mouse. Further, the ultrasonic transducer was fixed in the collimator on the head of each mouse. The isoflurane concentration was then adjusted to 0.7%–0.8%, which rendered the mice free of spontaneous movements. The conditions were held constant for 20 min before turning on the ultrasound stimulation, ensuring the same anaesthetic conditions for each animal. The experiment was terminated when the mouse returned its righting reflex or after two doses of 30 min US stimulation.

#### 4.3.3. Resistance to Anaesthesia Experiment

To examine the difference in the anaesthesia latency time between the sham and US groups, mice from both groups were treated with ultrasound or sham stimulation for 30 min while awake, then placed in the chamber with 1% isoflurane in oxygen. The time it took each mouse to lose its righting reflex was measured as the time of descent into anaesthesia.

#### 4.3.4. EC50 Experiment

Mice from both groups were pretreated with the corresponding stimulations for 30 min, then placed back into the anaesthesia chamber. The concentration of isoflurane was increased 0.2% every 20 min (0%, 0.2%, 0.4%, 0.6%, 0.8%, and 1.0%), and the number of mice without righting reflex was observed to calculate the fraction of LORR mice.

#### 4.3.5. Awakening from Anaesthesia Experiment

Both groups of mice received the corresponding stimulus while inhaling 1% isoflurane for 30 min. The mice were then returned to their home cages in room air. The latency time of restoring the righting reflex was recorded as the time to emergence.

#### 4.3.6. Antagonist Experiment

The behavior score and time to emergence were measured by the same protocol as in normal arousal experiments and emergence time experiments, while 1.0 ml vehicle (normal saline, i.p.) and the D1 receptor antagonist SCH-23390 (0.1 mg/kg, i.p.; Casmart) or D2 receptor antagonist (s)-(-)-sulpiride (50 mg/kg, 5 mg/kg, i.p.; Casmart) were administered after 20 min of inhalation at 2% isoflurane concentration.

#### 4.3.7. Arousal Scoring

The behaviors of the mice in the experiment were recorded using a video recorder. The person performing the scoring was unaware of our interventions in the experiment (e.g., ultrasound versus sham; saline versus SCH-23390/sulpiride; VTA versus V1) to prevent biasing. The awakening behaviors of the mice during the US stimulation phase were recorded and scored using an adaptation method [60]. The frequencies of head, whisker, leg, and tail movements were classified as none, mild, and moderate and scored as 0, 1, and 2, respectively. A score of 2 was assigned if the mouse landed on all four paws and 0 if it remained prone. The arousal response was based on the sum of all categories.

#### 4.3.8. Traumatic Brain Injury (TBI) Model in Mice

Different groups of mice were anaesthetized with isoflurane. The mice were depilated and scalped by conventional surgery to expose the skull. An impact point was marked at 2 mm near the left midline and 1 mm in front of the coronal suture. A 60 g cylindrical impact weight was allowed to free fall along a metal tube (RWD, 68093, China) with a vertical height of 13 cm, causing a depressed fracture of the skull [29]. Subsequently, 15 min of US stimulation was given to each injured mouse 15 min after injury; the awakening time was observed and recorded.

#### 4.3.9. Open Field Test

A 50 cm × 50 cm test box was used to perform the classic open field test to evaluate the motility of different groups of modeled mice. One hour after injury, each mouse was initially placed in the center of the box and allowed to explore freely for 10 minutes. The movement abilities of the mice were judged by analyzing the movement distances of the mice in the last 5 min using smart behavioral analysis software (SMART, Panlab, Spain).

### 4.4. Immunofluorescence

For double immunostaining of c-Fos and TH, mice were stimulated with ultrasound for 40 min and then perfused intracardially with 20 ml saline followed by 50 ml 4% PFA. The brains were harvested and fixed in 4% PFA solution for 4 h, followed by overnight equilibration with 30% sucrose in phosphate buffer at 4°C to dehydrate the brain. Finally, the VTA was sliced into 30 *μ*m brain slices along the coronal plane using a frozen slicer (CM1950, Leica, Germany).

Immunohistochemistry was performed using the free-floating method. Sections were washed in 0.1 M phosphate-buffered saline (PBS, pH 7.4) and incubated with 0.3% H_2_O_2_ for 15 min to quench endogenous peroxidase activity. After being washed in PBS, rabbit polyclonal primary antibody against c-Fos (1 : 10000, Ab5, Cat# PC38, Oncogene Research Products) was used to incubate the sections at room temperature in PBS containing 0.25% Triton-X-100 and 0.02% sodium azide. After being washed three times with PBS on the next day, biotinylated goat anti-rabbit IgG antibody (1 : 1000, BA-1000, Vector Laboratories) and avidin-biotin peroxidase complex ABC solution (1 : 1000, PK-6100, Vector Laboratories) were used for incubation 1 h. The peroxidase reaction was visualized with 0.05% 3,3′-diaminobenzidine tetrahydrochloride (Sigma, MO, USA) in PBS and 0.01% H_2_O_2_ and strengthened with 0.002% Ni and 0.001% CoCl_2_. The sections were then incubated overnight at room temperature with a rabbit polyclonal primary antibody against TH (1 : 10,000, TB700, Sigma). After termination with PBS-azide, the sections were mounted, dehydrated, and covered with slips. Adjacent sections were incubated in the absence of the primary antibody to confirm that no nonspecific staining had occurred. These images were taken with a slide-scanning microscope (VS120, Olympus, Japan).

### 4.5. Histology

To confirm the location and safety of US stimulation, we performed histological staining after the behavioral experiment. Fourteen mice were used for the safety test (sham: *n* = 6, ultrasound: *n* = 8). After anaesthesia, the brains were obtained and sections were taken using the method described previously. The formalin-fixed, paraffin-embedded brains were constantly sectioned into 8 *μ*m thick slices. Each brain was coronally sectioned into at least 5 slices. For H&E staining, sections were stained with haematoxylin solution for 10 min, followed by 5 dips in 1% acid ethanol for differentiation. The eosin solution was used to stain sections for 3 min. Then, the sections were dehydrated, cleared, and mounted in a resinous medium. One of the typical histological methods to visualize neurons in the brain is Nissl staining [61]. For Nissl staining, 0.5% thionine blue (G1032, Servicebio, China) was used for 5 min staining and 1% glacial acetic acid (G10000218, Servicebio, China) after washing with distilled water for differentiation. Xylene and resin medium were used to clear and mount the sections, respectively. A digital microscope slide scanner was utilized to observe the brain staining.

### 4.6. Statistical Analysis

Statistical comparisons were performed using independent-sample *t*-test and ANOVA (SPSS ver.13.0, IBM, USA) for the behavioral test results. All the data are presented as the mean ± SEM. The level of statistical significance was set at *p* ≤ 0.05. Statistical power of test (1 − *β*) was also evaluated by using G∗Power 3 (Version 3.1.9.7, Germany).

## Data Availability

All data are available in the manuscript or supplementary materials or from the author.

## Supplementary Materials

Supplementary 1 Supplementary Method: calculations of spatial-peak temporal-average intensity (ISPTA) and maximum temperature increasement. Figure S1: staining images of the VTA region in the sham and US groups. Figure S2: c-Fos expression in the locus coeruleus (LC) after ultrasound stimulation of the VTA. Figure S3: c-Fos expression in the periaqueductal grey (PAG) after ultrasound stimulation of the VTA. Figure S4: skull surface temperature before and after ultrasound stimulation. Figure S5: acoustic field distributions of the ultrasound. Table S1: behavioral responses of C57/BL-6 mice under general anaesthesia by VTA stimulation using ultrasound at 360 kPa. Table S2: behavioral responses of C57/BL-6 mice under general anaesthesia by VTA stimulation using ultrasound at 586 kPa. Table S3: behavioral responses of C57/BL-6 mice under general anaesthesia by VTA stimulation using ultrasound at 758 kPa. Table S4: behavioral responses of C57/BL-6 mice under SCH-23390 during ultrasound stimulation of the VTA. Table S5: behavioral responses of C57/BL-6 mice under sulpiride during ultrasound stimulation of the VTA. Table S6: behavioral responses of C57/BL-6 mice under general anaesthesia by VTA stimulation using ultrasound at the VTA and visual cortex.

Supplementary 2 Video S1: protocol of and representative behavioral performance in the normal behavior experiment for the sham and US groups.

Supplementary 3 Video S2: representative behavioral performance in the normal behavior experiment for the SCH-23390 and saline groups.

Supplementary 4 Video S3: representative behavioral performance in the normal behavior experiment or the sulpiride and saline groups.

Supplementary 5 Video S4: representative behavioral performance in the normal behavior experiment or the VTA and cortex groups.
