# Supplementary material for: Noninvasive Ultrasound Stimulation of Ventral Tegmental Area Induces Reanimation from General Anaesthesia in Mice
Source: Research (Wash D C). 2021 Apr 12;2021:2674692. doi: 10.34133/2021/2674692 (PMC8059556; doi:10.34133/2021/2674692)
Supplement: Supplementary 1 — Supplementary Method: calculations of spatial-peak temporal-average intensity (ISPTA) and maximum temperature increasement. Figure S1: staining images of the VTA region in the sham and US groups. Figure S2: c-Fos expression in the locus coeruleus (LC) after ultrasound stimulation of the VTA. Figure S3: c-Fos expression in the periaqueductal grey (PAG) after ultrasound stimulation of the VTA. Figure S4: skull surface temperature before and after ultrasound stimulation. Figure S5: acoustic field distributions of the ultrasound. Table S1: behavioral responses of C57/BL-6 mice under general anaesthesia by VTA stimulation using ultrasound at 360 kPa. Table S2: behavioral responses of C57/BL-6 mice under general anaesthesia by VTA stimulation using ultrasound at 586 kPa. Table S3: behavioral responses of C57/BL-6 mice under general anaesthesia by VTA stimulation using ultrasound at 758 kPa. Table S4: behavioral responses of C57/BL-6 mice under SCH-23390 during ultrasound stimulation of the VTA. Table S5: behavioral responses of C57/BL-6 mice under sulpiride during ultrasound stimulation of the VTA. Table S6: behavioral responses of C57/BL-6 mice under general anaesthesia by VTA stimulation using ultrasound at the VTA and visual cortex. [file 2674692.f1.docx]

**Supplementary Materials**

**Ultrasound stimulation of ventral tegmental area induces reanimation from general anaesthesia in mice**

Tianyuan Bian^1,#^, Wen Meng^1,#^, Meihong Qiu^2^, Zhigang Zhong^2^, Zhengrong Lin^1^, Junjie Zou^1^, Yibo Wang^1^, Xiaowei Huang^1^, Lisheng Xu^3^, Tifei Yuan^4^, Zhili Huang^2^, Lili Niu^1,*^, Long Meng^1,*^, Hairong Zheng^1^,^*^

**^1^ Institute of Biomedical and Health Engineering, Shenzhen Institutes of Advanced Technology, Chinese Academy of Sciences, 1068 Xueyuan Avenue, Shenzhen, China, 518055**

**^2^ Department of Pharmacology, School of Basic Medical Sciences, Fudan University, Shanghai, China, 200032**

**^3^ College of Medicine and Biological Information Engineering, Northeastern University, 195 Innovation Road, Shenyang 110016, China.**

**^4^ Shanghai Mental Health Center, Shanghai Jiaotong University School of Medicine, Shanghai, China, 200030.**

**^#^ These authors contributed equally to this work and are co-first authors**

***Correspondence should be addressed to Lili Niu; lili.niu@siat.ac.cn, Long Meng;** [**long.meng@siat.ac.cn**](mailto:long.meng@siat.ac.cn)**, and Hairong Zheng;** [**hr.zheng@sita.ac.cn**](mailto:hr.zheng@sita.ac.cn)

**Supplementary Method: Calculations of spatial-peak temporal-average intensity (I_SPTA_) and maximum temperature increasement.**

Ultrasound intensity parameter measurement system, including a calibrated needle hydrophone (SN2791, 0.5 mm probe, Precision acoustics, Dorchester, UK) and a 3D ultrasound intensity measurement system (UMS3, Precision acoustics, Dorchester, UK), was used to measure the acoustic field parameters without and with mouse skull as previously described. When scanning the XY plane, the acoustic pressure map of 0.8×0.8 mm was scanned with 0.02 mm steps when the hydrophone found the maximum value of X, Y and Z axis. When scanning the YZ plane, the acoustic pressure map was scanned by spanning 0.8×4 mm with 40 steps both direction.

The focal length of the transducer is about 5 mm, and the focus is 4.5 mm away from the skull surface when the designed collimator was worn, which exactly cover the VTA area.

The mechanical index (MI) was defined as $\text{MI=}\frac{\text{P}_{\text{r}}}{\sqrt{\text{f}}}$, where $\text{P}_{\text{r}}$ is the derated peak negative pressure of the ultrasound wave in MPa and f is the frequency of the ultrasound in MHz. The pulse pressure squared integral (PPI) was defined as

$\text{PPI=}\int_{\text{t}_{0}}^{\text{t}_{0}\text{+nT}} \text{p}^{2}\text{(t)dt}$,

where $\text{p}\left(\text{t}\right)$ is the instantaneous pressure, T is the period of the ultrasound, n is the number of complete period of the selected waveform, and $\text{t}_{0}$ is the latency time of the first full amplitude period.

The pulse intensity integral (PII) is defined as

$\text{PII=}\int_{\text{t}_{0}}^{\text{t}_{0}\text{+nT}} \frac{\text{p}^{2}\text{(t)}}{\text{ρc}}\text{dt}\text{=}\text{PPI}/\text{ρc}$,

where $\text{ρ}$ is the density of water, c is the speed of the ultrasound in water.

The spatial peak pulse average intensity ($\text{I}_{\text{SPTA}}$) was calculated as

$\text{I}_{\text{SPPA}}\text{=}\frac{1}{\text{nT}}\int_{\text{t}_{0}}^{\text{t}_{0}\text{+nT}} \frac{\text{p}^{2}\text{(t)}}{\text{ρc}}\text{dt}\text{=}\frac{\text{PII}}{\text{TBD}}$,

where TBD stands for tone burst duration.

The spatial peak time average intensity ($\text{I}_{\text{SPTA}}$) is calculated as

$\text{I}_{\text{SPTA}}\text{=I}_{\text{SPPA}}\times\text{DC=MAX}\left[\text{PII}\left(\text{z}\right)/\text{T}_{\text{PRF}}\right]\text{=PII}\times\text{PRF}$,

where DC is the duty cycle, $\text{T}_{\text{PRF}}$ is the time interval between pulses and PRF is the pulse repetition frequency.

In our study, the $\text{I}_{\text{SPPA}}$and $\text{I}_{\text{SPTA}}$without skull are 7.94 W/cm^2^ and 4.03 W/cm^2^, 1.40 W/cm^2^ and 719.04 mW/cm^2^ with skull. Acoustic pressure decreased about 50.4% after passing through the skull, and the focusing situation had a little change in the shape and penetration depth. In addition, the tissue rising temperature was estimated by the formula as:

$\text{ΔT=}\frac{\text{QΔt}}{\text{C}_{\text{v}}}$,

where $\text{C}_{\text{v}}$ is the heat capacity per unit volume for tissue defined as:

$\text{C}_{\text{v}}\text{=Cρ}$,

where C is the heat capacity in tissue, $\text{C=3.6}\text{J}/\text{g}/^{\circ}\text{C}$ for brain tissue, and $\text{ρ}$ is the density of the tissue, $\text{ρ=1028}\text{kg}/\text{m}^{3}$for brain tissue.

The heat generation per volume (Q) is defined as:

$\text{Q=2αI}_{\text{SPTA}}$,

where $\text{α}$ is the absorption coefficient in tissue, $\text{α}$is less than $0.\text{5Np}/\text{cm}$at 3.3 MHz. The tissue rising temperatures of skull and brain were calculated by the above method. For VTA, the result of rising temperature is 0.1$^{\circ}C$.


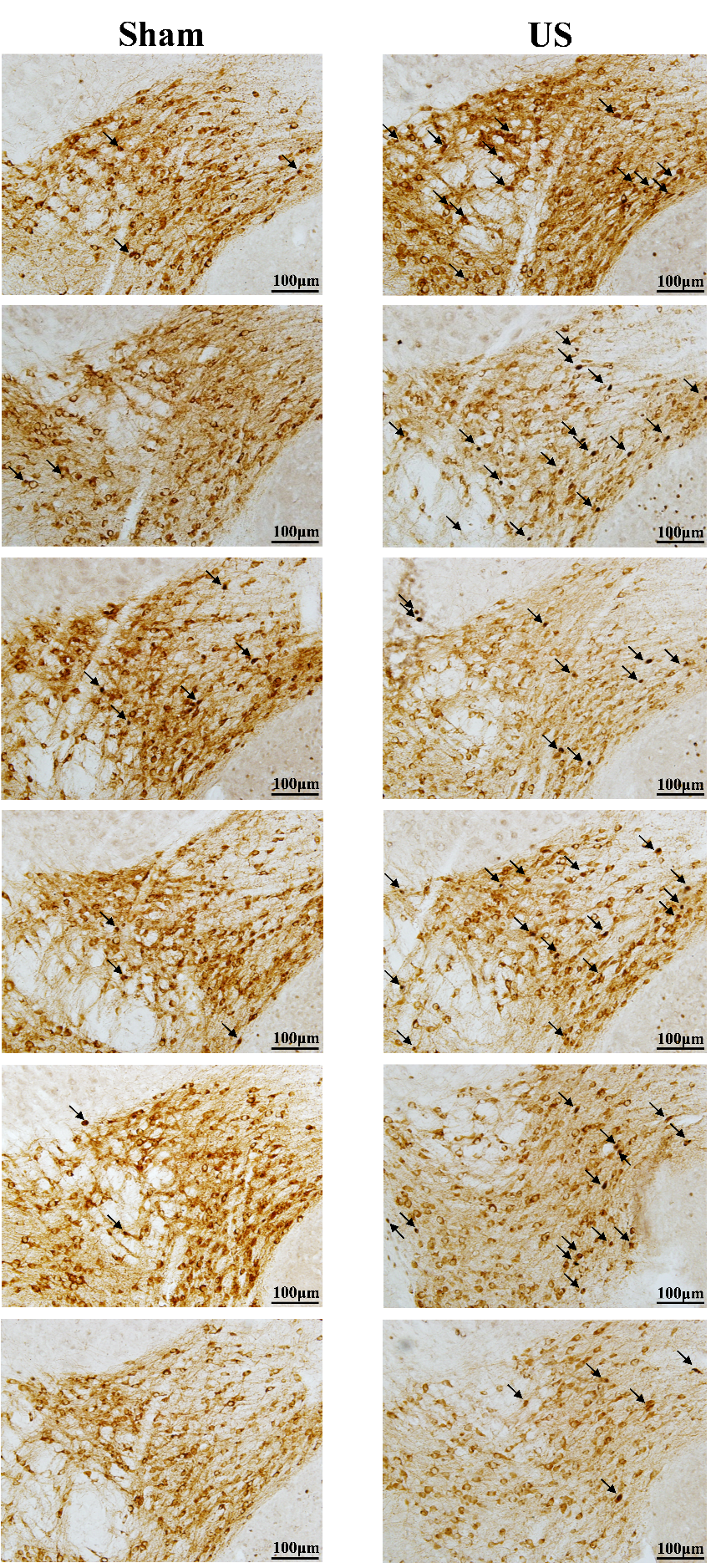


**Fig. S1. Staining images of the VTA region in the sham and US groups.** Double labelling of TH (brown) and c-Fos (black) in the VTA. Arrows indicate c-Fos-positive cells. The results showed that ultrasound stimulation could significantly increase the number of c-Fos-positive cells in the VTA region.


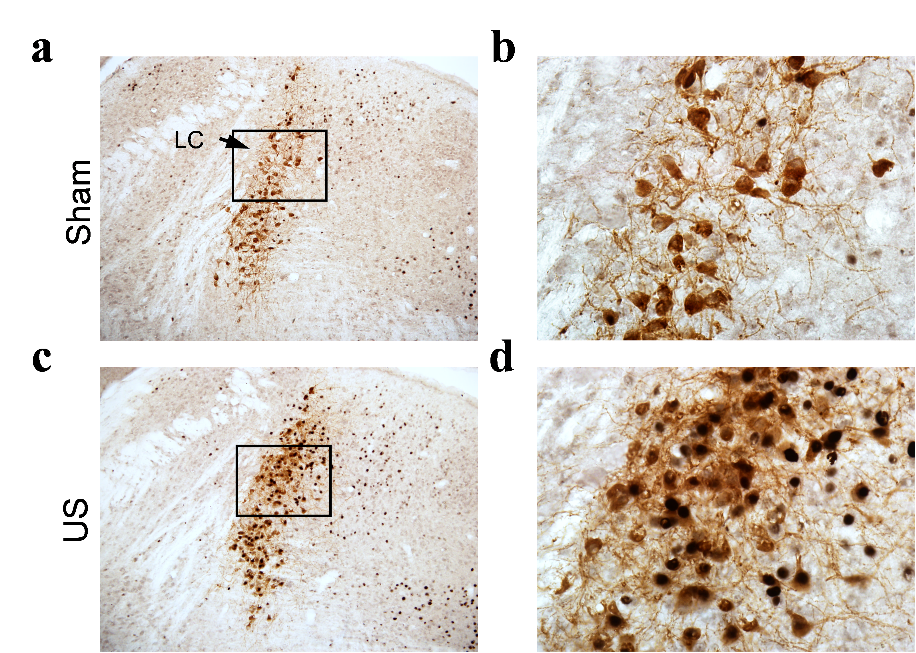


**Fig. S2. C-Fos expression in the locus coeruleus (LC) after ultrasound stimulation of the VTA.** Representative c-Fos staining from LC area in Sham group (**a, b**) and US group (**c, d**). Double labeling of TH (brown) and c-Fos (black) staining in the LC. Black spots indicate the c-Fos positive cells in figure **b, d**.


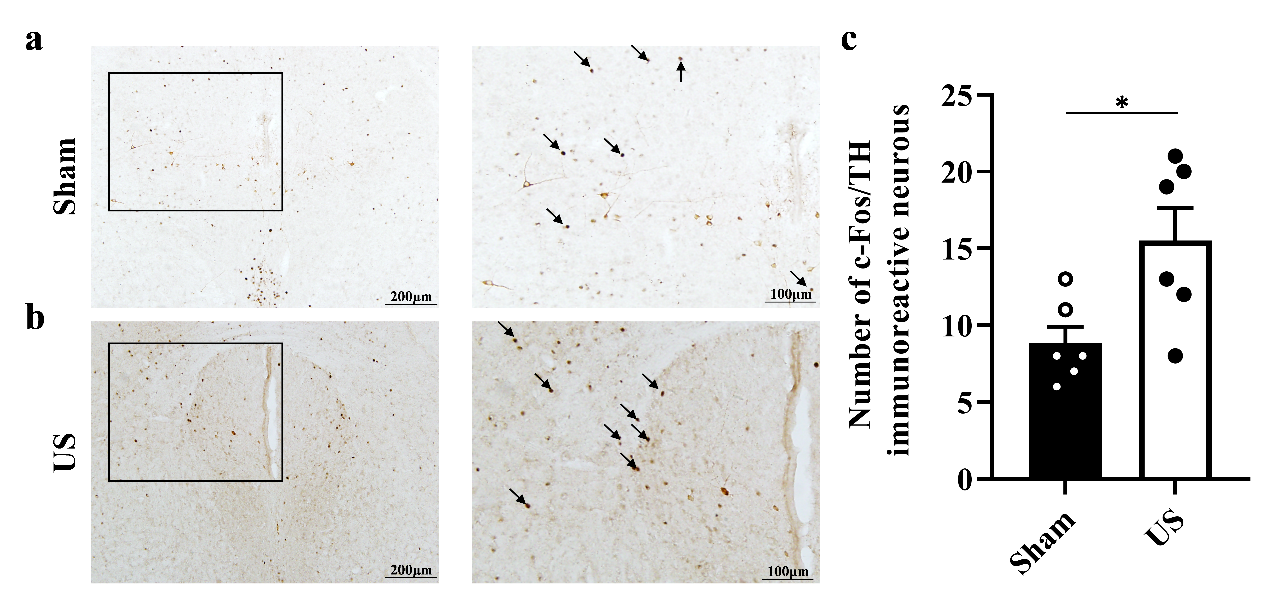


**Fig. S3. C-Fos expression in the periaqueductal grey (PAG) after ultrasound stimulation of the VTA.** Ultrasound stimulation affects the abundance of c-Fos-positive neurons in the PAG. Representative c-Fos (black) expression in PAG in sham group (a) and US group (b). Arrows indicate c-Fos-positive cells. (c), Small increase in the number of c-Fos-positive cells in the US group compared to the sham group (n = 6 for the US group and n = 6 for the sham group, mean ± SEM, *p < 0.05, independent samples t-test).

**
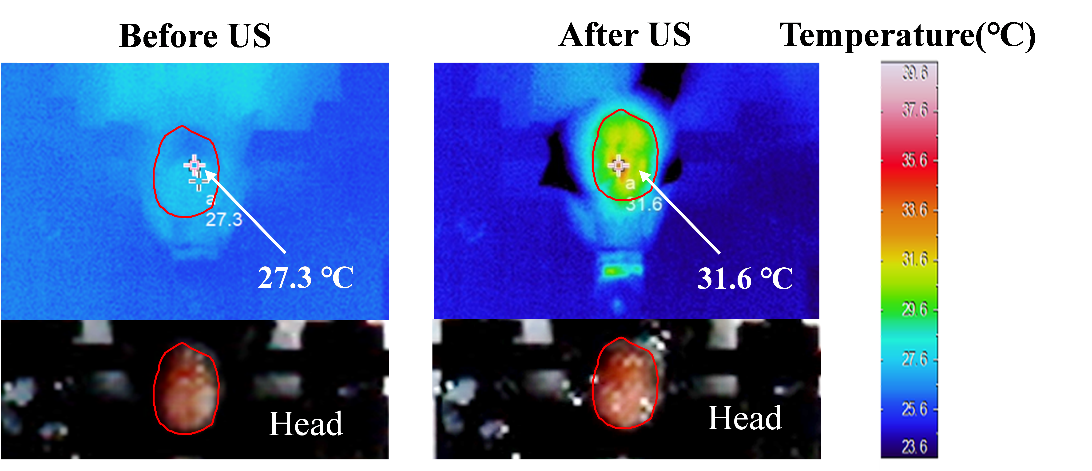
**

**Fig. S4. Skull surface temperature before and after ultrasound stimulation.** The surface temperature of the skull were measured by an infrared thermal imager (R300, NEC Avio, Tokyo, Japan).


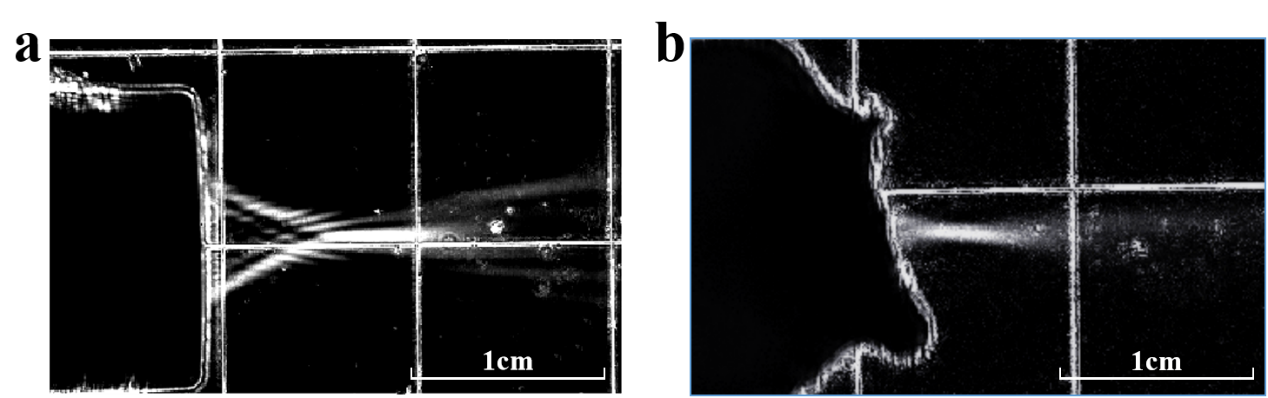


**Fig. S5. Acoustic field distributions of the ultrasound.** Acoustic field distributions in longitudinal plane **a,** without and **b,** with mouse skull were measured by the OptiSon® Ultrasound Beam Analyzer (Onda, USA).

**Table S1: Behavioral responses of C57/BL-6 mice under general anaesthesia by VTA stimulation using ultrasound at 360 kPa.**

| Group | Ultrasound  /Sham | Leg movement | Head movement | Whisker movement | Tail movement | Righting | Total score |
| --- | --- | --- | --- | --- | --- | --- | --- |
| 1 | Ultrasound | 1 | 0 | 0 | 1 | 0 | 2 |
|  | Sham | 0 | 0 | 0 | 1 | 0 | 1 |
| 2 | Ultrasound | 1 | 0 | 1 | 1 | 0 | 3 |
|  | Sham | 0 | 0 | 0 | 0 | 0 | 0 |
| 3 | Ultrasound | 0 | 0 | 1 | 1 | 0 | 2 |
|  | Sham | 0 | 0 | 0 | 1 | 0 | 1 |
| 4 | Ultrasound | 1 | 0 | 0 | 1 | 0 | 2 |
|  | Sham | 0 | 0 | 0 | 1 | 0 | 1 |
| 5 | Ultrasound | 1 | 0 | 0 | 2 | 2 | 3 |
|  | Sham | 0 | 0 | 0 | 0 | 0 | 0 |
| 6 | Ultrasound | 1 | 0 | 0 | 1 | 0 | 2 |
|  | Sham | 1 | 0 | 0 | 0 | 0 | 1 |
| 7 | Ultrasound | 1 | 0 | 0 | 1 | 0 | 2 |
|  | Sham | 0 | 0 | 0 | 1 | 0 | 1 |
| 8 | Ultrasound | 1 | 0 | 0 | 1 | 0 | 2 |
|  | Sham | 0 | 0 | 1 | 0 | 0 | 1 |

The arousal scoring system was adapted from Reed et al. Leg, head, and whisker movements were scored as absent, mild, or moderate in intensity (0, 1, or 2, respectively) during 30 min of ultrasound VTA stimulation while mice continuously inhaled 0.7 to 0.8% isoflurane. Righting was scored as 0 if the mouse remained prone, and 2 if all four paws touched the ground.

**Table S2: Behavioral responses of C57/BL-6 mice under general anaesthesia by VTA stimulation using ultrasound at 586 kPa.**

| Group | Ultrasound  /Sham | Leg movement | Head movement | Whisker movement | Tail movement | Righting | Total score |
| --- | --- | --- | --- | --- | --- | --- | --- |
| 1 | Ultrasound | 2 | 1 | 1 | 2 | 2 | 8 |
|  | Sham | 1 | 0 | 0 | 0 | 0 | 1 |
| 2 | Ultrasound | 2 | 2 | 1 | 2 | 2 | 9 |
|  | Sham | 1 | 0 | 0 | 1 | 0 | 2 |
| 3 | Ultrasound | 2 | 1 | 2 | 2 | 0 | 7 |
|  | Sham | 0 | 0 | 0 | 0 | 0 | 0 |
| 4 | Ultrasound | 1 | 1 | 2 | 2 | 2 | 8 |
|  | Sham | 0 | 0 | 1 | 0 | 0 | 1 |
| 5 | Ultrasound | 1 | 2 | 1 | 2 | 2 | 8 |
|  | Sham | 0 | 1 | 1 | 0 | 0 | 2 |
| 6 | Ultrasound | 2 | 2 | 1 | 2 | 0 | 7 |
|  | Sham | 0 | 0 | 0 | 0 | 0 | 0 |
| 7 | Ultrasound | 2 | 1 | 1 | 2 | 2 | 8 |
|  | Sham | 1 | 0 | 0 | 0 | 0 | 1 |
| 8 | Ultrasound | 2 | 2 | 1 | 1 | 2 | 8 |
|  | Sham | 0 | 0 | 0 | 0 | 0 | 0 |

The arousal scoring system was adapted from Reed et al. Leg, head, and whisker movements were scored as absent, mild, or moderate in intensity (0, 1, or 2, respectively) during 30 min of ultrasound VTA stimulation while mice continuously inhaled 0.7 to 0.8% isoflurane. Righting was scored as 0 if the mouse remained prone, and 2 if all four paws touched the ground.

**Table S3: Behavioral responses of C57/BL-6 mice under general anaesthesia by VTA stimulation using ultrasound at 758 kPa.**

| Group | Ultrasound  /Sham | Leg movement | Head movement | Whisker movement | Tail movement | Righting | Total score |
| --- | --- | --- | --- | --- | --- | --- | --- |
| 1 | Ultrasound | 2 | 1 | 2 | 2 | 2 | 9 |
|  | Sham | 1 | 0 | 1 | 0 | 0 | 2 |
| 2 | Ultrasound | 2 | 1 | 1 | 1 | 2 | 7 |
|  | Sham | 0 | 0 | 0 | 1 | 0 | 1 |
| 3 | Ultrasound | 2 | 1 | 2 | 2 | 0 | 7 |
|  | Sham | 0 | 0 | 0 | 1 | 0 | 1 |
| 4 | Ultrasound | 1 | 1 | 2 | 2 | 2 | 8 |
|  | Sham | 0 | 0 | 1 | 0 | 0 | 1 |
| 5 | Ultrasound | 1 | 2 | 1 | 2 | 2 | 8 |
|  | Sham | 0 | 1 | 0 | 1 | 0 | 2 |
| 6 | Ultrasound | 2 | 2 | 1 | 2 | 0 | 7 |
|  | Sham | 0 | 0 | 0 | 1 | 0 | 1 |
| 7 | Ultrasound | 2 | 2 | 1 | 2 | 0 | 7 |
|  | Sham | 1 | 0 | 0 | 1 | 0 | 2 |
| 8 | Ultrasound | 2 | 2 | 1 | 1 | 2 | 8 |
|  | Sham | 0 | 0 | 1 | 1 | 0 | 2 |

The arousal scoring system was adapted from Reed et al. Leg, head, and whisker movements were scored as absent, mild, or moderate in intensity (0, 1, or 2, respectively) during 30 min of ultrasound VTA stimulation while mice continuously inhaled 0.7 to 0.8% isoflurane. Righting was scored as 0 if the mouse remained prone, and 2 if all four paws touched the ground.

**Table S4: Behavioral responses of C57/BL-6 mice under SCH-23390 during ultrasound stimulation of the VTA**

| Group | Drug | Leg movement | Head movement | Whisker movement | Tail movement | Righting | Total score |
| --- | --- | --- | --- | --- | --- | --- | --- |
| D1  (0.1mg/kg) | NS-US | 2 | 2 | 1 | 2 | 2 | 9 |
|  | SCH-23390 US | 1 | 1 | 0 | 1 | 0 | 3 |
|  | SCH-23390 Sham | 1 | 0 | 0 | 2 | 0 | 3 |
| D1  (0.1mg/kg) | NS-US | 2 | 1 | 2 | 2 | 0 | 7 |
|  | SCH-23390 US | 0 | 0 | 1 | 2 | 0 | 3 |
|  | SCH-23390 Sham | 0 | 0 | 0 | 1 | 0 | 1 |
| D1  (0.1mg/kg) | NS-US | 1 | 1 | 1 | 2 | 2 | 7 |
|  | SCH-23390 US | 1 | 0 | 0 | 1 | 0 | 2 |
|  | SCH-23390 Sham | 0 | 0 | 0 | 1 | 0 | 1 |
| D1  (0.1mg/kg) | NS-US | 2 | 2 | 1 | 2 | 2 | 9 |
|  | SCH-23390 US | 0 | 0 | 0 | 2 | 0 | 2 |
|  | SCH-23390 Sham | 0 | 0 | 0 | 2 | 0 | 2 |
| D1  (0.1mg/kg) | NS-US | - | - | - | - | - | - |
|  | SCH-23390 US | 1 | 0 | 0 | 1 | 0 | 2 |
|  | SCH-23390 Sham | 0 | 0 | 1 | 1 | 0 | 2 |
| D1  (0.1mg/kg) | NS-US | - | - | - | - | - | - |
|  | SCH-23390 US | 0 | 0 | 0 | 1 | 0 | 1 |
|  | SCH-23390 Sham | 0 | 0 | 0 | 1 | 0 | 1 |

The arousal scoring system was adapted from Reed et al. Leg, head, and whisker movements were scored as absent, mild, or moderate in intensity (0, 1, or 2, respectively) during 30 min of ultrasound VTA stimulation while mice continuously inhaled 0.7 to 0.8% isoflurane. Righting was scored as 0 if the mouse remained prone, and 2 if all four paws touched the ground.

**Table S5: Behavioral responses of C57/BL-6 mice under Sulpiride during ultrasound stimulation of the VTA**

| Group | Drug | Leg movement | Head movement | Whisker movement | Tail movement | Righting | Total score |
| --- | --- | --- | --- | --- | --- | --- | --- |
| D2  (50mg/kg) | NS-US | 1 | 2 | 1 | 2 | 0 | 6 |
|  | Sulpiride US | 1 | 2 | 0 | 1 | 2 | 6 |
|  | Sulpiride Sham | 0 | 0 | 1 | 2 | 0 | 3 |
| D2  (50mg/kg) | NS-US | 1 | 1 | 2 | 2 | 0 | 6 |
|  | Sulpiride US | 2 | 2 | 1 | 2 | 0 | 7 |
|  | Sulpiride Sham | 0 | 0 | 0 | 0 | 0 | 0 |
| D2  (50mg/kg) | NS-US | 1 | 1 | 1 | 2 | 0 | 5 |
|  | Sulpiride US | 2 | 2 | 0 | 2 | 0 | 6 |
|  | Sulpiride Sham | 1 | 0 | 0 | 0 | 0 | 1 |
| D2  (50mg/kg) | NS-US | 1 | 2 | 2 | 1 | 0 | 6 |
|  | Sulpiride US | 1 | 1 | 1 | 2 | 0 | 5 |
|  | Sulpiride Sham | 1 | 0 | 0 | 1 | 0 | 2 |
| D2  (50mg/kg) | NS-US | - | - | - | - | - | - |
|  | Sulpiride US | 2 | 1 | 1 | 2 | 2 | 8 |
|  | Sulpiride Sham | 0 | 0 | 0 | 1 | 0 | 1 |
| D2  (50mg/kg) | NS-US | - | - | - | - | - | - |
|  | Sulpiride US | 1 | 1 | 1 | 2 | 0 | 5 |
|  | Sulpiride Sham | 0 | 1 | 1 | 0 | 0 | 2 |
| D2  (5mg/kg) | NS-US | 2 | 1 | 2 | 1 | 2 | 8 |
|  | Sulpiride US | 0 | 1 | 2 | 2 | 0 | 5 |
|  | Sulpiride Sham | 0 | 0 | 0 | 1 | 0 | 1 |
| D2  (5mg/kg) | NS-US | 1 | 1 | 1 | 2 | 0 | 5 |
|  | Sulpiride US | 1 | 0 | 2 | 2 | 0 | 5 |
|  | Sulpiride Sham | 0 | 1 | 1 | 0 | 0 | 2 |
| D2  (5mg/kg) | NS-US | 1 | 1 | 2 | 1 | 2 | 7 |
|  | Sulpiride US | 2 | 1 | 1 | 1 | 2 | 7 |
|  | Sulpiride Sham | 0 | 0 | 0 | 1 | 0 | 1 |
| D2  (5mg/kg) | NS-US | 2 | 1 | 1 | 2 | 2 | 8 |
|  | Sulpiride US | 2 | 2 | 1 | 2 | 2 | 9 |
|  | Sulpiride Sham | 0 | 0 | 1 | 0 | 0 | 1 |
| D2  (5mg/kg) | NS-US | 2 | 1 | 2 | 1 | 0 | 6 |
|  | Sulpiride US | 2 | 1 | 1 | 1 | 0 | 5 |
|  | Sulpiride Sham | 0 | 0 | 0 | 1 | 0 | 1 |
| D2  (5mg/kg) | NS-US | 1 | 2 | 2 | 1 | 2 | 8 |
|  | Sulpiride US | 1 | 1 | 0 | 1 | 2 | 5 |
|  | Sulpiride Sham | 1 | 0 | 1 | 0 | 0 | 2 |

The arousal scoring system was adapted from Reed et al. Leg, head, and whisker movements were scored as absent, mild, or moderate in intensity (0, 1, or 2, respectively) during 30 min of ultrasound VTA stimulation while mice continuously inhaled 0.7 to 0.8% isoflurane. Righting was scored as 0 if the mouse remained prone, and 2 if all four paws touched the ground.

**Table S6: Behavioral responses of C57/BL-6 mice under general anaesthesia by VTA stimulation using ultrasound at the VTA and visual cortex.**

| Group | VTA/Cortex | Leg movement | Head movement | Whisker movement | Tail movement | Righting | Total score |
| --- | --- | --- | --- | --- | --- | --- | --- |
| 1 | VTA | 1 | 2 | 1 | 2 | 2 | 8 |
|  | Cortex | 1 | 1 | 0 | 2 | 0 | 4 |
| 2 | VTA | 1 | 2 | 1 | 1 | 2 | 7 |
|  | Cortex | 1 | 1 | 0 | 1 | 0 | 3 |
| 3 | VTA | 2 | 2 | 1 | 2 | 2 | 9 |
|  | Cortex | 0 | 0 | 0 | 2 | 0 | 2 |
| 4 | VTA | 1 | 1 | 2 | 2 | 2 | 8 |
|  | Cortex | 1 | 0 | 1 | 2 | 0 | 4 |
| 5 | VTA | 2 | 2 | 1 | 2 | 2 | 9 |
|  | Cortex | 0 | 1 | 1 | 0 | 2 | 4 |
| 6 | VTA | 2 | 2 | 1 | 2 | 0 | 7 |
|  | Cortex | 1 | 1 | 0 | 1 | 0 | 3 |

The arousal scoring system was adapted from Reed et al. Leg, head, and whisker movements were scored as absent, mild, or moderate in intensity (0, 1, or 2, respectively) during 30 min of ultrasound stimulation while mice continuously inhaled 0.7 to 0.8% isoflurane. Righting was scored as 0 if the mouse remained prone, and 2 if all four paws touched the ground.
